# Hippocampal and Cortical Primary Cilia Are Required for Aversive Memory in Mice

**DOI:** 10.1371/journal.pone.0106576

**Published:** 2014-09-03

**Authors:** Nicolas F. Berbari, Erik B. Malarkey, S. M. Zaki R. Yazdi, Andrew D. McNair, Jordyn M. Kippe, Mandy J. Croyle, Timothy W. Kraft, Bradley K. Yoder

**Affiliations:** 1 Department of Cell, Developmental, and Integrative Biology, The University of Alabama at Birmingham, Birmingham, Alabama, United States of America; 2 Department of Vision Sciences, The University of Alabama at Birmingham, Birmingham, Alabama, United States of America; Technion - Israel Institute of Technology, Israel

## Abstract

It has been known for decades that neurons throughout the brain possess solitary, immotile, microtubule based appendages called primary cilia. Only recently have studies tried to address the functions of these cilia and our current understanding remains poor. To determine if neuronal cilia have a role in behavior we specifically disrupted ciliogenesis in the cortex and hippocampus of mice through conditional deletion of the Intraflagellar Transport 88 (*Ift88*) gene. The effects on learning and memory were analyzed using both Morris Water Maze and fear conditioning paradigms. In comparison to wild type controls, cilia mutants displayed deficits in aversive learning and memory and novel object recognition. Furthermore, hippocampal neurons from mutants displayed an altered paired-pulse response, suggesting that loss of IFT88 can alter synaptic properties. A variety of other behavioral tests showed no significant differences between conditional cilia mutants and controls. This type of conditional allele approach could be used to distinguish which behavioral features of ciliopathies arise due to defects in neural development and which result from altered cell physiology. Ultimately, this could lead to an improved understanding of the basis for the cognitive deficits associated with human cilia disorders such as Bardet-Biedl syndrome, and possibly more common ailments including depression and schizophrenia.

## Introduction

Nearly every cell in the body possesses a primary cilium, a small microtubule based appendage now known to serve as a signaling hub for a diverse set of pathways [1]. The use of model systems such as *Chlamydomonas reinhardtii* and *Caenorhabditis elegans* has revealed much about how cilia are assembled through a highly conserved process known as intraflagellar transport (IFT) and disrupting ciliary proteins in neurons has been shown to impact behavior in *C. elegans* (for a review see [2]). However, whether primary cilia have any role in the neurophysiology of mammalian neurons has not been well described. Collectively, the research done in these model systems and more recently in mouse mutants and human patients with ciliary defects have revealed that the primary cilium is a highly complex sensory and signaling center throughout both embryonic development and for adult tissue homeostasis [3].

Clinically, a growing list of human genetic syndromes, termed ciliopathies, has been identified as having cilia dysfunction as their root cause [4]. Multiple ciliopathies (e.g. Bardet-Biedl syndrome, Alström syndrome, Joubert syndrome) present with both neurodevelopmental and cognitive deficits. Furthermore, cilia perturbation in mouse models can lead to neurodevelopmental phenotypes including cerebellar malformations, altered neuronal migration, and defects in adult neurogenesis [5]–[8]. The phenotypes observed in these mouse models closely mimic the clinical features observed in human ciliopathy patients. As such mouse models are very important tools for understanding the cellular and physiological underpinnings responsible for the cognitive and behavioral deficits in ciliopathy patients

Another possible connection between cilia and regulation of behavior was made by Einstein *et al*. who demonstrated that disruption of the ciliary localized G-Protein Coupled Receptor somatostatin receptor 3 (Sstr3) caused deficits in novel object recognition [9]. However, a direct role for cilia in this process was not evaluated. Here, we extend this analysis by analyzing the behavioral and cognitive consequences of disrupting IFT/cilia in the hippocampus and cortex of the mouse brain.

## Results

### Ift88^Δ/Δ^;Emx1-Cre mutants have cilia loss specific to the hippocampus and cortex

**Figure 1:**
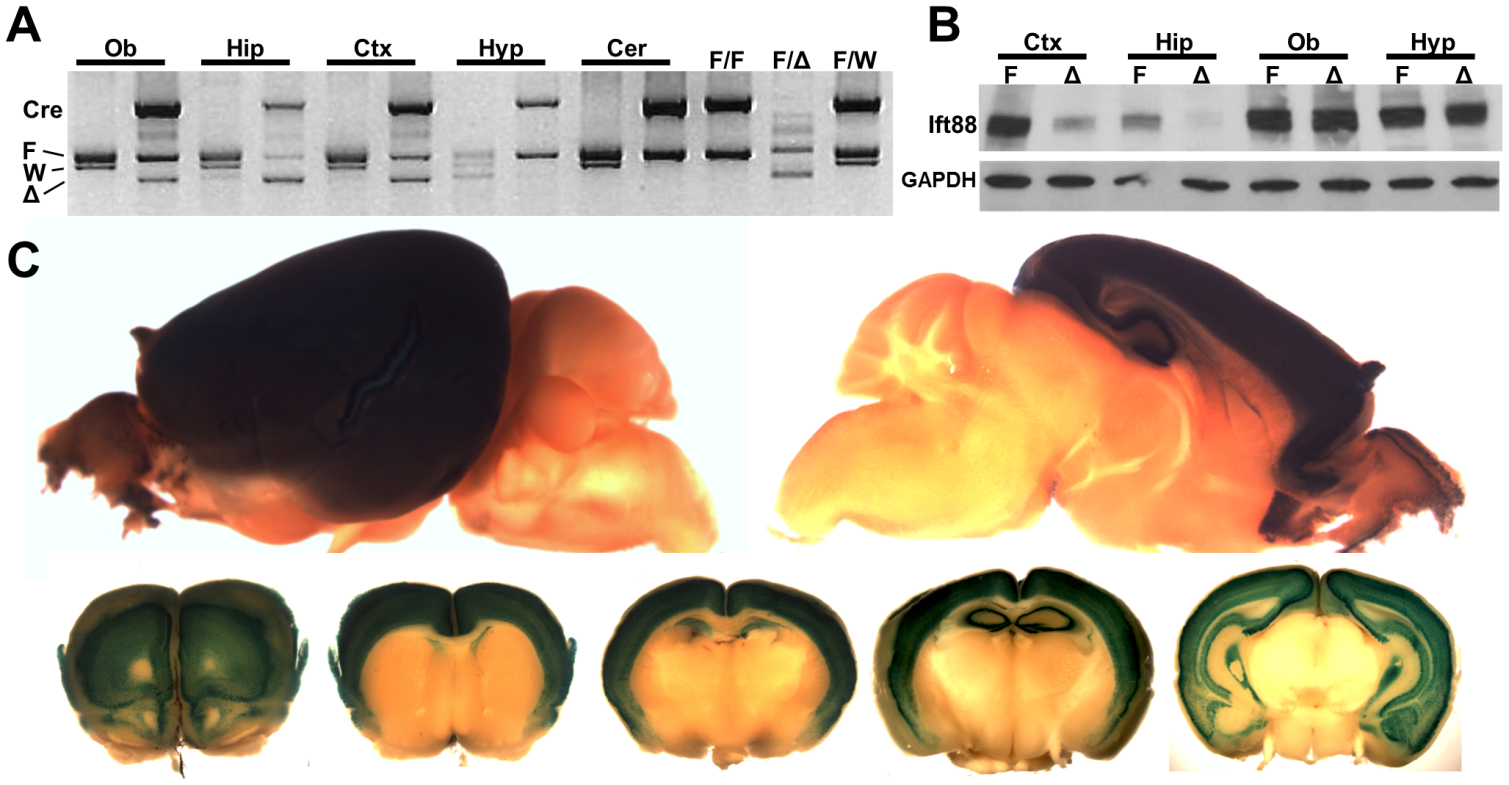
Conditional loss of IFT88 in the adult hippocampus and cortex. (**A**) PCR genotyping from whole genomic DNA prepped from the following brain regions: olfactory bulb (Ob), hippocampus (Hip), cortex (Ctx), hypothalamus (Hyp), and cerebellum (Cer). Bands from top to bottom are as follows: Emx1-cre transgene (cre), Ift88 floxed conditional allele (F), wildtype allele (W), mutant allele (Δ). Note the induction of the mutant band (Δ) when Emx1-cre (cre) occurs only in Ob, Hip, and Ctx and not in Hyp and Cer. Control samples from genomic DNA isolated from ear punches are as follows: *Ift88^flox/flox^*; Emx1-cre (F/F), *Ift88^flox/Δ^* (F/Δ), *Ift88^flox/wildtype^*; Emx1-cre (F/W). (**B**) Immunoblotting from whole proteins isolated from Ctx, Hip, Ob, and Hyp probed for Ift88 and a loading control Gapdh. Control samples from *Ift88^flox/flox^* (F) are next to experimental *Ift88^Δ/Δ^*; Emx1-cre (Δ). Note the diminished levels of Ift88 in Δ samples only occurs in ctx and hip. (**C**) Cre reporter lacZ staining of whole mount brains shows the regions of cre activity. Top row shows a sagittal half and the bottom row shows transverse sections with most rostral to caudal from left to right.

To determine the effects of hippocampal and cortical cilia loss on behavior we utilized a previously described conditional allele of a gene required for cilia formation and maintenance, *Intraflagellar transport 88* (hereafter referred to as *Ift88^flox/flox^*) [10]. The *Ift88^flox/flox^* mice were crossed with mice carrying the transgene *Empty spiracles homeobox 1* promoter driven Cre recombinase (hereafter referred to as *Emx1-Cre*) where Cre is expressed specifically in the telencephalon, [11]. To confirm the specificity of the Cre line and the efficacy of Ift88 loss, we used PCR, immunoblotting, Cre reporter analysis, and immunofluorescence microscopy. Region specific PCR and Western blotting revealed that the mutant allele (hereafter *Ift88^Δ/Δ^*) and IFT88 protein loss occurred only in the olfactory bulb, hippocampus, and cortex but not in control regions such as the hypothalamus and cerebellum (Figure **1A and B**).

**Figure 2:**
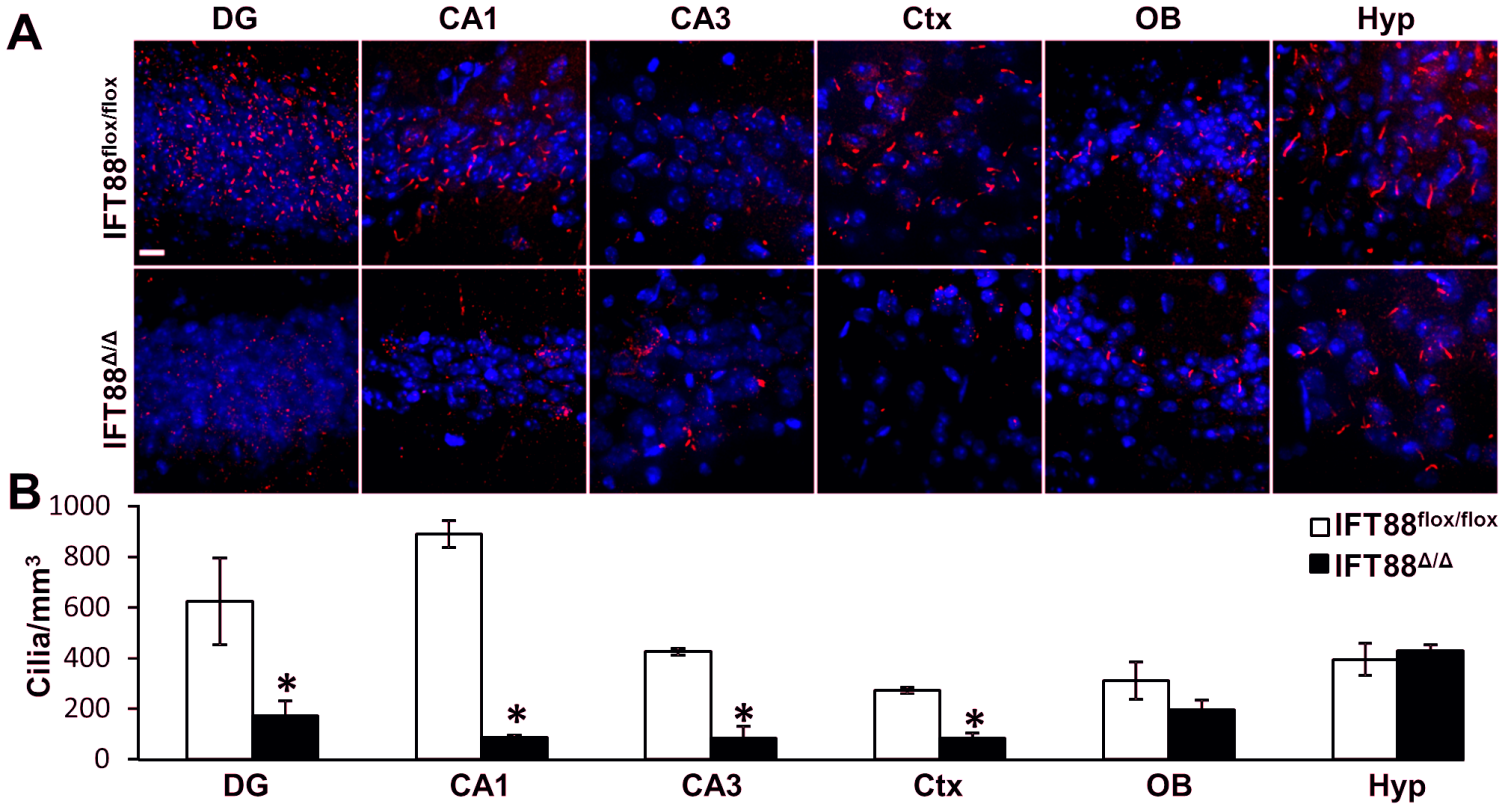
Conditional loss of Cilia in the adult hippocampus and cortex. (**A**) Immunofluorescence for the neuronal cilia marker adenylate cyclase III (red) in brain sections from the following regions of the brain: Ob, Ctx, and regions of the hippocampus the pyramidal layers CA1, CA3, and granule layer of the dentate gyrus (DG). *Ift88^flox/flox^* control samples (top row) are compared to *Ift88^Δ/Δ^*; Emx1-cre (bottom row). Hoechst nuclear stain is in blue. Scale bar is 50 µm. (**B**) Graph indicating the amount of cilia counted in several regions of the brain in control (*Ift88^flox/flox^*, N = 3) and mutants (*Ift88^Δ/Δ^* N = 4). Statistically significant differences are indicated: Student's t-test, * p<0.05.

Emx1-Cre specificity was further confirmed using a Rosa26 Cre reporter that expresses β-galactosidase only in cells in which Cre was active (Figure **1C** **and File S1**) [12]. Direct analysis of cilia using antibodies to the cilia marker adenylate cyclase III (ACIII) showed a marked reduction in cilia number occurred only in the regions of Cre expression (Figure **2A, B** **and File S2, S3, S4**) [13]. We further confirmed that the Emx1-Cre was not promiscuously expressed or associated with other phenotypes associated with early neurodevelopmental roles of cilia. For example, in the eye photoreceptor degeneration has been observed in Bbs mutants [14]. To rule out the presence of vision deficits in *Ift88^Δ/Δ^;Emx1-Cre* mutants electrorentinogram analysis was performed and the dark adapted threshold b-wave, and maximal b-wave were not different between the wild type and mutant animals; likewise the light adapted a- and b-waves were also not different (File **S5**). We subsequently performed genotyping by PCR on whole eye DNA to test directly for the presence of the mutant allele (File **S5**). These results indicate that adult *Ift88^Δ/Δ^*; Emx1-Cre mutants were not visually impaired. Upon establishing the spatial specificity and efficacy of the Cre to delete Ift88 and cause cilia loss, we assessed whether there are behavioral phenotypes in *Ift88^Δ/Δ^* mutants.

### Ift88^Δ/Δ^; Emx1-Cre mutants have deficits in aversive and recognition memory

**Figure 3:**
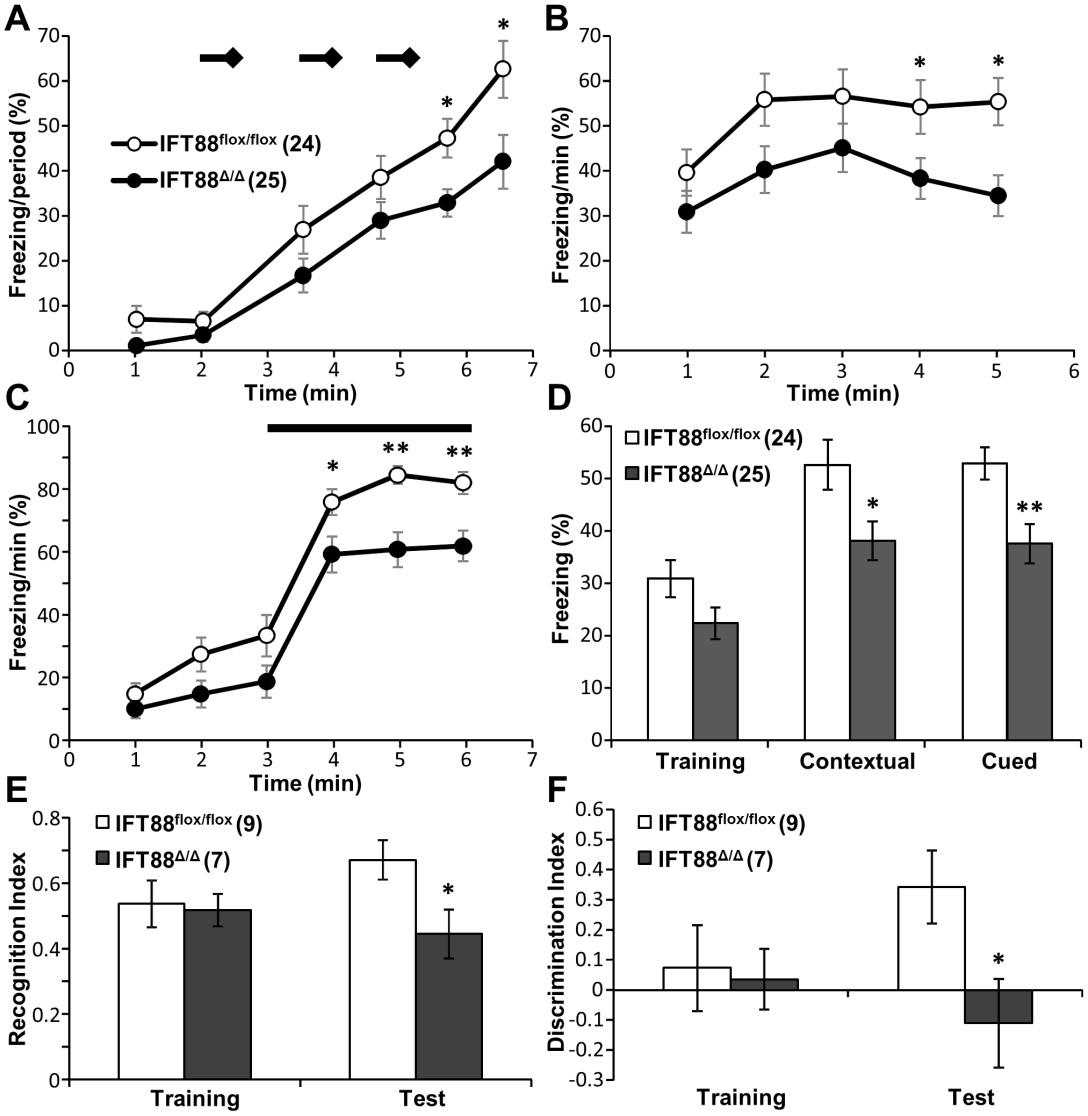
Fear conditioning and novel object recognition in mice lacking hippocampal and cortical primary cilia. (**A**) Percent of time spent freezing during training on day 1, bars and diamonds represent tones and shocks respectively (**B**) Day 2: Percent time spent freezing during the contextual test in same arena. (**C**) Day 3: Percent time freezing during the cued test in new arena, bar represents when tone was played. (**D**) Comparison of freezing time between cilia mutant and wild type mice. Mutant mice spent less time freezing. (**E**) Novel object recognition index as the time spent investigating the novel object relative to the total object investigation and (**F**) discrimination index as the exploration time devoted to the novel object minus the time devoted to the familiar object. Statistically significant differences are indicated: Student's t-test, * p<0.05, ** p<0.01.

We tested aversive learning and memory in *Ift88^Δ/Δ^; Emx1-Cre* mice using contextual and cued fear conditioning paradigms. Interestingly, mutants displayed a baseline decrease in freezing after foot shock during the last minutes of the training phase (Figure **3A**). There was also a decrease in mutant freezing on the testing day in both the contextual and cued fear conditioning compared to controls (Figure **3B, C, and D**). Similarly, *Ift88^Δ/Δ^; Emx1-Cre* mutants displayed a deficit in both recognition and discrimination indices in the novel object recognition assay suggesting a role for hippocampal and cortical cilia in recognition memory (Figure **3E and F**). These results show that Ift88 in neurons of the cortex and hippocampus in the adult mouse has a role in establishing certain types of memory (aversive and recognition). This could be a direct effect of loss of IFT itself or secondary effects caused by the loss of the cilium.

### Ift88^Δ/Δ^;Emx1-Cre mutants do not have spatial memory, anxiety, nociception or motor deficits

**Figure 4:**
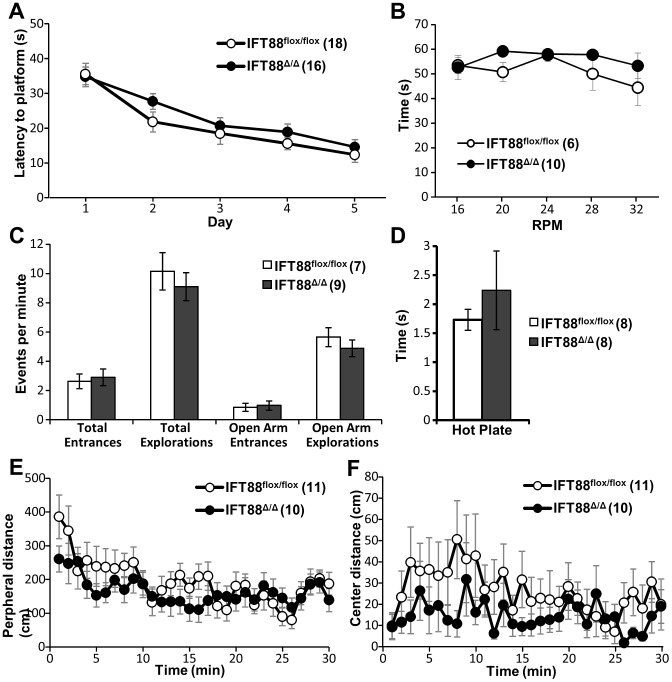
Behavior testing in mice lacking primary cilia in hippocampus and cortex. (**A**) Morris Water Maze displayed as latency to platform in seconds (s) during the experimental day. (**B**) RotaRod test results displays as time on rod in seconds (s) at specific speeds in rotations per minute (RPM). (**C**) Elevated plus maze displayed in events per minute for explorations and entrances (**D**) Hotplate test displaying time in seconds till reaction (s) (**E** and **F**) Open field test result displayed in distance in centimeters (cm) from the chamber periphery (**E**) and center (**F**). Control (Ift88^flox/flox^) and mutant (Ift88^Δ/Δ^) mice are indicated throughout figure. Animal numbers are indicated in parenthesis next to group. No statistically significant differences in any behavioral test were determined using Student's t-test.

The difference we observed in the fear conditioning paradigm indicated the potential for a hippocampal defect; therefore we tested spatial memory using the Morris Water Maze (MWM) which showed no differences between mutants and controls (Figure **4A**). Since we saw a phenotype in fear conditioning and none in MWM one possible explanation would be that our mutant mice had an overall change in anxiety related behaviors. We tested this possibility by employing both the elevated plus and open field paradigms and observed no differences between mutants and controls (Figure **4C, D and F**). Another possible explanation for the fear conditioning result would be potential changes in nociception in the mutant mice where they would be less sensitive to the foot shock and thus spend less time freezing. Furthermore, Bbs mouse models and patients have been reported to have changes in thermosensation and nociception [15]. To test this possibility we utilized the hotplate test and found no differences in mutants and controls (Figure **4D**). To determine if there is change in motor cortex function we used rotorod and found no differences (Figure **4B**). These combined results suggest that cilia in the hippocampus and cortex are not involved in MWM associated spatial learning and memory, anxiety, nociception or coordination (Figure 4**A, B, and D**).

### Ift88^Δ/Δ^; Emx1-Cre mutants brains have electrophysiological deficits

**Figure 5:**
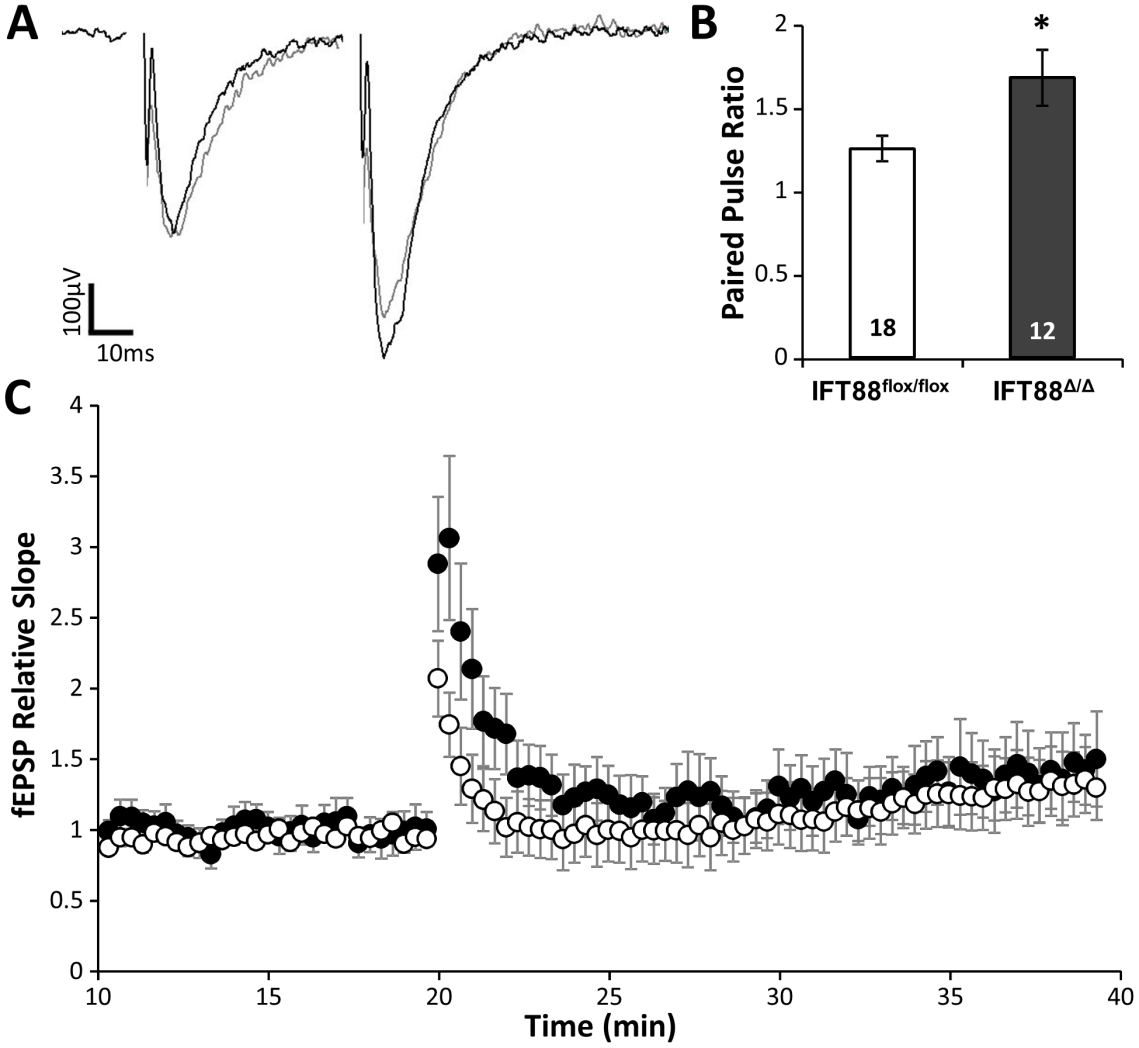
Altered paired-pulse facilitation in mice lacking hippocampal and cortical primary cilia. Field recordings from CA1 hippocampus after schaffer collateral stimulation. (**A**) example traces of paired fEPSPs recorded in wild type (white) and mutant (black) hippocampal slices. (**B**) Cilia mutant neurons show an increase in paired pulse facilitation. (**C**) Long term potentiation recordings after 4 100 hz trains. Statistically significant differences are indicated: Student's t-test, * p<0.05.

To determine if there was a neurophysiological change that would account for the deficits in object recognition and fear responses in the mutant mice, we performed field recordings from the CA1 region of hippocampal slices while stimulating the Schaffer collaterals in CA3. *Ift88^Δ/Δ^; Emx1-Cre* mutants showed no change in induction of long term potentiation compared to wild type controls after 100 hz stimulation. However, we did observe a 40% increase in the paired pulse facilitation of mutant mice (Figure **5A, B, and C**). These data show that loss of an IFT protein can influence the presynaptic transmitter release from hippocampal neurons.

## Discussion

Some of the first evidence indicating that neuronal cilia may play a role in signaling came from the observation that several G-protein coupled receptors (GPCRs), such as somatostatin receptor 3 (Sstr3) and serotonin receptor 6 (Htr6), preferentially localize to the membrane of the cilium in certain regions of the brain [16], [17]. Moreover, several GPCRs do not properly localize to neuronal cilia in mouse models of the ciliopathy, Bardet-Biedl Syndrome (BBS) [18]–[20]. Interestingly, human BBS patients frequently exhibit mental deficits and behavioral issues such as low IQ and depression [21], [22]. The list of receptors and disease associated proteins that localize to neuronal cilia continues to grow, however, the relevance of this localization and the physiological roles for cilia in the brain remain unknown [23]–[25].

One complication with analyzing cilia function in adult neuronal physiology is that in both mutant mouse models and human ciliopathy patients there are neuronal development phenotypes and clinical features arising from congenital loss of cilia function. For example Bbs4 knockout mice display neuronal defects in a region specific manner. The work by Agassandian showed a loss of ACIII staining in specific brain regions such as the hippocampus and amygdala but not in other regions [26]. This led the authors to suggest that perhaps defects in cilia of these brain regions result in some of the neuropathological deficits associated with BBS. Interestingly, earlier work by the same group showed in the arcuate nucleus of the hypothalamus of Bbs2 and Bbs6 knockout mice that there was a significant loss of proopiomelanocortin neurons [27]. However, neuronal loss was not proposed to be the cause of the obesity phenotype in these mice. These studies using congenital mouse ciliopathy models indicate the importance of conditional approaches to address roles for cilia in specific CNS regions.

One study looking at neuronal cilia function using conditional alleles in the CNS comes from work by Amador-Arjona et al. [28]. In this work the authors analyze cilia in the CNS after development using a conditional allele of Intraflagellar transport protein 20. However, one concern from this study is that the Cre line used would result in cilia loss in all GFAP expressing cells and descendants, including astrocytes, oligodendrocytes and neurons and thus specific claims about neuronal cilia are limited. In agreement with the work presented here using an Emx1-Cre to ablate cilia on neurons these authors also observed a novel object recognition phenotype.

In our cre reporter analysis and cilia immunofluorescence indicate prominent and specific Cre expression in both the cortex and hippocampus. It is interesting to note that in the Emx1-Cre *Ift88^Δ/Δ^* mutants the dentate gyrus has noticeably less cilia than its control counterpart, yet some cilia are still present. It is possible that these remaining cilia are simply derived from cells that do not express Emx1 or that there is a degree of incomplete expression of the Cre in these regions of the brain. In fact there are certain populations of neurons such as the cortical GABAergic lineage which have been shown not to express Emx1 [29]. Another possible reason for these remaining cilia in the dentate is that the adult neurogenic niche in the sub-granular zone remains intact in this Emx1-Cre conditional approach, which may populate the hippocampus with ciliated neurons [30]. It is enticing to hypothesize that these cilia in the mutant dentate gyrus are allowing mice to perform normally in a spatial memory paradigm such as the MWM. Perhaps the near complete loss of cilia in other regions of the hippocampus is enough to show deficits in both the aversive and recognition memory tasks of fear conditioning and novel object recognition. It is also of note that the mutant olfactory bulb showed Cre induction but also remains somewhat ciliated. This observation is also in line with the possibility that adult neurogenesis in this model is intact, particularly the sub-ventricular zone adult neurogenic niche. Interestingly, mutant mice do not have overt changes in olfaction as they are capable of finding hidden food in the same amount of time as controls (data not shown). It is unclear if the adult neural precursors in our model have been affected and future studies will focus specifically on how cilia perturbation may alter the process of adult neurogenesis.

Another novel finding regarding the neurophysiological effects of IFT loss was seen in field recordings of the CA1 of the hippocampus. While there was no overt effect on long term potentiation, there was a clear phenotype in paired pulse facilitation. This result implicates primary cilia are involved in regulating the synaptic activity of hippocampal and cortical neurons. Surprisingly, this change in paired pulse facilitation indicates that this effect relates to pre-synaptic release of neurotransmitter. It remains to be determined if this is a direct role for the cilium, or a secondary effect of cilia loss. In other systems, such as in the kidney, primary cilia have been shown to be intimately involved in regulation of Ca^2+^ signaling [31]–[33]. One possibility is that the loss of cilia on presynaptic neurons may result in increased presynaptic Ca^2+^ concentration leading to a greater release probability. However, how the primary cilium at the cell body would exert an influence at the axon terminals is not clear. Another possibility is that upon loss of IFT, the molecular motors that are responsible for cilia formation ectopically redistribute along the microtubules of neuronal processes and have subsequent effects on neuronal physiology. Regardless of the precise molecular mechanism behind this result, it is interesting that effects on cilia may directly alter neuronal physiology.

Recently Disrupted-in-Schizophrenia-1 (DISC1) and many other genes implicated in psychiatric disorders were found to have effects on primary cilia when knocked down *in vitro* [24], [25]. In a broader sense it is interesting that the DISC-1 gene product has also been implicated in cilia biology. For example DISC1 localizes to the base of the cilium at the centrosome and was found to interact with pericentriolar material 1 (PCM1) and the Bardet-Biedl syndrome protein 4 (Bbs4) [34]. In another example, human microtubule-interacting protein associated with TRAF3 (MIP-T3/IFT54) is required for DISC-1 localization as well as for formation of cilia [35], [36]. Together these data suggest that perturbations in primary cilia may play unrecognized roles in psychiatric disorders. It is also likely that cilia located on neurons in other regions of the brain may impact behavior. A prime example where cilia have been implicated in regulating behavior comes from studies of hypothalamic neurons and control of feeding behavior. When cilia are lost from hypothalamic proopiomelanocortin (POMC) neurons of mice, they become hyperphagic and obese [37]. Thus, these data combined with the results presented here suggest that conditional loss of cilia in other regions of the brain will reveal additional unrecognized roles for cilia in behavior. A better understanding of cilia in the central nervous system could yield valuable insights into a wide range of ciliopathies as well as more common disorders such as dementia and schizophrenia [38].

## Materials and Methods

### Mice

To generate cohorts, females homozygous for the *Ift88^flox^* conditional allele (*Ift88^tm1Bky^*) were crossed with Emx1-Cre transgenic (Tg(Emx1-cre)5Yql); homozygous *Ift88^flox^* mice to yield either experimental *Ift88^flox/flox^*; Emx1-cre mice or gender matched control *Ift88^flox/flox^* [10], [11].

### Ethics Statement

All mice in this study were maintained on an inbred C57BL/6 genetic background and experimental procedures were approved by the Institutional Animal Care and Use Committee (IACUC) regulations at the University of Alabama at Birmingham under animal protocol number (130208061).

### Tissue Preparation

Mice were anesthetized by a 0.1 ml per 10 g of body weight intraperitoneal injection of 2.5% tribromoethanol (Sigma-Aldrich, Saint Louis, MO, USA), killed by cardiac puncture, and perfused with PBS followed by 4% paraformaldehyde (Fisher Scientific, Pittsburgh, PA, USA). The brains were then immersion fixed in 4% paraformaldehyde overnight at 4°C followed by cryoprotection in 30% sucrose in PBS overnight at 4°C. Cryoprotected brains were embedded in Optimal Cutting Temperature compound (Fisher Scientific) and cryosectioned at 20 µm.

### Cre Activity Reporter Analysis

A transgenic β-galactosidase cre reporter (Gt(ROSA)26Sor^tm1Sor^) [12] was crossed to Emx1-cre and whole-mount X-Gal staining was performed to assess regions of cre activity. Briefly, brains were collected and cut sagittal down the midline or in 3 millimeter transverse sections and then fixed in 2% paraformaldehyde for 30 minutes on ice. After washing in *lacZ* buffer (2 mM MgCl2, 0.01% NaDC, 0.02% NP40, in 100 mM sodium phosphate buffer, pH 7.3), tissues were incubated at 37°C in 1 mg/ml X-Gal diluted in 5 mM potassium ferrocyanide and 5 mM potassium ferricyanide [39].

### Immunoblotting

Whole hypothalami were dissected and isolated into ice-cold lysis buffer (137 mM NaCl, 20 mM Tris pH 8.0, 1% Triton X-100, 10% glycerol, and complete EDTA-free protease inhibitor cocktail (Roche Diagnostics, Indianapolis, IN, USA)). After a 5 second sonication the tissue was incubated on ice for 30 minutes and then vortexed briefly before centrifugation at 10,000×g at 4°C for 10 minutes. Protein concentrations were determined by Bradford assay (Bio-Rad Laboratories, Hercules, CA, USA). Protein samples were resolved on a denaturing 10% Tris-HCL gel (Bio-Rad Laboratories) and transferred to Immobilon-Psq transfer membrane (Millipore, Billerica, MA, USA). Membranes were blocked in TBS-T (10 mM Tris-HCl pH 7.5, 150 mM NaCl, 0.1% Tween-20) with 5% milk for 1 hour and incubated with primary antibody diluted in TBS-T with 2% BSA for 16–24 hours at 4°C. Membranes were probed with horseradish peroxidase (HRP) conjugated secondary antibodies diluted in TBS-T with 1% milk for 1 hour at room temperature. Secondary antibodies were detected using SuperSignal West Pico Chemiluminescent Substrate (Pierce/Thermo Scientific, Waltham, MA, USA) and bands were visualized using Blue Ultra Autorad Film (Bioexpress ISC Kaysville, UT, USA). The following primary antibodies and dilutions were used: anti-actin (A2066; Sigma-Aldrich) 1∶1,000, anti-Adenylate cyclase III (sc-588; Santa Cruz Biotechnology, Santa Cruz, CA, USA) 1∶500, and anti-Ift88 rabbit polyclonal 1∶500 (Yoder Laboratory [40]). Secondary antibodies were HRP conjugated anti-rabbit (#31460) 1∶5,000, HRP conjugated anti-mouse (#31430) 1∶10,000 (Pierce/Thermo Scientific).

### Immunofluorescence

Brain sections were washed twice for 5 minutes in PBS before being permeabilized with 0.3% Triton X-100 in PBS with 2% donkey serum, 0.02% sodium azide and 10 mg/ml bovine serum albumin (BSA). Primary antibody incubations were performed for 16–24 hours at 4°C and secondary antibody incubations performed for 1 hour at room temperature. Primary antibody was anti-adenylyl cyclase III (ACIII) (1∶500, Santa Cruz Biotechnology, Santa Cruz CA). Secondary antibody was Alexa Fluor-546 conjugated donkey anti-rabbit IgG (Invitrogen, Carlsbad, CA). Nuclei were visualized by Hoechst nuclear stain (Sigma-Aldrich). Sections were cover slipped and mounted with DABCO (Sigma-Aldrich).

### Confocal Microscopy and Image Analysis

All fluorescence images were captured on Perkin Elmer ERS 6FE spinning disk confocal microscope and images were processed and analyzed in Volocity version 6.1.1 software (Perkin Elmer, Shelton, CT, USA). Cilia were counted by detecting all objects with ACIII intensity greater than 7 SD above the mean image intensity that were between 1 and 10 µm^3^. All images were acquired using the same camera exposure, gain and laser power settings.

### Electroretinography

Mice were dark-adapted overnight prior to measuring ERGs. Animals were anesthetized via intraperitoneal injection of xylazine (9.09 mg/kg) and ketamine (90.9 mg/kg). Corneas were anesthetized with proparacaine (0.5%) and one pupil dilated with topical phenylephrine HCl (2.5%) and tropicamide (1%). During recording the animal was placed in a Faraday cage with head fixed by a bite-bar and body temperature maintained at 38°C by a heating pad (Braintree Scientific, Braintree, MA). A 100-W tungsten-halogen lamp acted as the light source and was focused onto one end of a fiber optic. A shutter with 6-mm aperture (Uniblitz; Vincent Associates, Rochester, NY) set the stimulation duration for 2 ms. Full-field ERGs were recorded using a, 2 mm diameter platinum wire loop embedded in the tapered end of a Plexiglas rod hollowed out to receive the fiber optic. This arrangement ensured a constant distance between the fiber optic and the eye; the tapered end also acts as a diffusing element, yielding an isotropic plane of illumination at the pupil. The reference electrode is a second platinum loop touching the non-stimulated eye. Methylcellulose (Goniosol, CIBA Vision Corp, Duluth, GA) is applied to both eyes as well as to the recording and reference corneal electrodes. The stimulus set consisted of persentations of 505 nm light beginning at threshold and proceeding in 0.6 log unit steps to the maximum output of our optical bench. After performing a series of flashes in the dark, a background light is presented for 3 minutes and bright flashes again presented to measure the light-adapted ERG response.

### Open Field

Mice were placed in an open field activity test chamber (ENV-515, Med Associates) measuring 17″ by 17″ and were allowed to roam freely for 30 minutes. Activity Monitor (Med Associates) was used to examine locomotor activity within the apparatus. The mice were then returned to their home cages and the boxes were cleaned with 70% ethanol. Data was analyzed using Activity MDB to Excel Converter (Med Associates) and Microsoft Excel.

### RotaRod

#### Training (Days 1–3)

*Day1*: Mice were placed on a RotaRod apparatus (ENV-575M, Med Associates) accelerating from 2.5–25 rpm for 60 seconds. Animals were removed from the apparatus and returned to their home cage for 5 minutes. An additional 3 trials were performed at a set speed of 24 rpm, 60 seconds max. In each trial the latency to fall was recorded. The apparatus was cleaned with 70% ethanol between each test.

*Days 2&3*: As on Day 1 four 60 second trials were performed at a set speed, 24 rpm.

#### Testing (Day 4)

Mice were tested by performing 2 trials at the following speeds: 5, 16, 20, 24, 28 and 32 rpm. The mice were returned to their home cage and allowed to recover 5 minutes between each trial and the apparatus was cleaned with 70% ethanol. Latency to fall was recorded.

### Elevated Plus

The mice were placed in an elevated plus maze containing 2 open arms and 2 closed arms and were allowed to roam freely for 1 minute. Time spent in each arm was recorded using Video Maze (Med Associates). The apparatus was cleaned with 70% ethanol between each trial.

### Hot Plate

Mice were placed on a 55°C hot plate and covered with a transparent glass beaker. Latency to mice jumping on the sides of the beaker was recorded.

### Fear Conditioning

#### Training (Day 1: Afternoon)

Mice between the ages of 10 and 20 weeks were placed one at a time in a chamber located within a larger box. The chamber had a shock plate for the floor. The box included a fan, light and speaker. A video camera placed on the door observed the entire arena. Two chambers were utilized, one of which contained patterned wallpaper lining the interior walls of the apparatus. Mice were placed in the chamber for 6.5 minutes each. During each trial, a tone played for 30 seconds and was followed by a 0.5 mA foot shock to the mouse. The tone and subsequent shock repeated two additional times. The chamber was cleaned using ethanol between each trial. No data was collected during the conditioning phase. Freezing time was measured in both subsequent test trials (see below).

#### Contextual Test (Day 2: Morning)

Mice were placed into the chambers for observation over a 5 minute period. The context of the box remained the same for each animal as it was for their conditioning. The cameras were calibrated before each trial to ensure accurate motion tracking. Video Freeze (Med Associates, St. Albans, VT USA) software was utilized to track the mice and analyze the percentage of time spent freezing. The camera recorded at 30 fps and mice were classified as exhibiting freezing behavior once minimum freeze duration of 30 frames had been achieved. No sound was played during the 5 minute test. The chamber was cleaned using ethanol between each trial [41].

#### Cued Test (Day 2: Afternoon)

Opaque plates were placed on the floor of the chamber as well as diagonally in the chamber so that the rectangular arena was halved. Additionally, the fans were turned off, isopropanol was used for cleaning, and peppermint oil was added to the chamber. Animals exposed to the wallpaper in conditioning were placed in the chamber lacking wallpaper. Video Freeze (Med Associates) software was utilized to track the mice and analyze the percentage of time spent freezing over each 6 minute trial. The camera recorded at 30 fps, with minimum freeze duration of 30 frames. During each trial, a tone played for 30 seconds, but no foot shock was administered.

### Morris Water Maze

A circular pool with a depth of 30 cm and diameter of 130 cm was filled with water and white tempura paint was added until water became opaque. A 10 cm^2^ platform was placed just below the water's surface in the northeast quadrant. Attached to the outside of each side of the tank was a wooden pole on which one of the following patterned shapes was mounted: rectangle, diamond, triangle, or circle. An initial trial was conducted with a flag on top of the platform to signify its location. Following this, four 60 second trials were conducted daily for five consecutive days. Noldus Ethovision XT tracking software was used to record the latency to find the platform, as well as velocity and path taken. Mice that failed to find the platform after 60 seconds were placed on the platform for 5 seconds. Mice were then gently placed in the pool by hand at the extreme of one cardinal direction. All four directions were used each day in a random order as the starting position. Daily means (including all four trials) were computed for each mouse and overall mutant vs. wild-type performance assessed on each day.

### Novel Object Recognition

On the first day mice were habituated in the experimental chamber for 10 minutes. Over the next four days the mice were placed into the chamber with two objects and allowed to explore for 10 minutes while being recorded with a video camera. On the fourth day one of the objects was replaced with a novel object. The third day of familiar objects was compared to the day of novel object testing. Analysis of mouse association with objects was performed using Noldus Ethovision XT 8.5 where mice body centroids approaching within 10 cm of an object was considered associating. Recognition Index (RI) was calculated as previously described using the following formula RI  =  *T*_N_/(*T*_N_ + *T*_F_) where *T*_N_ is the time spent with the novel object and *T*_F_ is time spent with the familiar object [42]. Discrimination Index (DI), was calculated using the following formula DI  =  (*T*_R_ −*T*_L_)/(*T*_R_ + *T*_L_) for when the objects are identical in the familiarization phase. *T*_R_ represents the exploration time devoted to the right sample and T_L_ represents the exploration time devoted to the left sample [42]. Thus a negative DI score indicates more time spent with the familiar object.

### Electrophysiology

Animals were anesthetized with isoflurane and decapitated. Cerebral hemispheres were quickly removed and placed into cold sucrose-ACSF (in mM: 75 sucrose, 87 NaCl, 2.5 KCl, 21.4 NaHCO3, 1.25 NaH2PO4, 0.5 CaCl2, 7 MgCl2, 1.3 ascorbic acid, 20 glucose) that was aerated with a 95% O2/5% CO2 mixture. Transverse hippocampal slices (400 µm thick) from wild-type and mutant mice (male or female, 8–12 weeks old) were cut with a Leica VT1000s (Leica Instruments, Nussloch, Germany) and transferred to a holding chamber to rest at room temperature for 1 h before transferring to a heated (37°C) recording chamber. Oxygenated ACSF (in mM: 75 sucrose, 87 NaCl, 2.5 KCl, 21.4 NaHCO3, 1.25 NaH2PO4, 0.5 CaCl2, 7 MgCl2, 1.3 ascorbic acid, 20 glucose) was continuously perfused through the chamber at a rate of 1–2 ml/min. Recordings were conducted after a 1 hr recovery period. A twisted pair electrode was used to stimulate the Schaffer collateral afferent fibers in the stratum radiatum of the hippocampal CA1 region. Field EPSP synaptic responses were sampled at 0.05 Hz using a test pulse with an intensity that produced a half-maximal response. Responses were recorded with low resistance glass microelectrodes (1–10 MΩ) filled with ACSF. LTP was induced by giving two tetanic trains (100 Hz, 200 ms duration) of 20 pulses with an interpulse interval of 5 s. Potentiation was determined by normalizing the fEPSP slope recorded after tetanic stimulation by the average of the baseline fEPSP slope. Traces were sampled and digitized using a Digidata 1200 (Axon Instruments) and collected using an Axopatch 200 b (Axon Instruments).

### Statistical methods and analysis

Statistics were analyzed using Microsoft excel. Differences between controls and mutant mouse groups in all behavioral tests and electrophysiological recordings were evaluated using Student's t-test. Normality was assessed using the Shapiro–Wilk test.

## Supporting Information

File S1 **Cre reporter lacZ staining of brains sections shows the regions of cre activity.** **(A)** Sagittal 20 µm thick brain section of the cortex (Ctx) and hippocampus (Hipp) stained for cre reporter LacZ activity in blue. **(B)** Image of the hippocampus showing the CA1, CA3 and dentate gyrus (DG). **(C)** Image of the cortex (Ctx) and CA1 of the hippocampus.
(TIF)

File S2 **Conditional loss of Cilia in the adult cortex.** Immunofluorescence for the neuronal cilia marker adenylate cyclase III (red) in the cortex (Ctx). **(A and B)** Stitching of several 20X images such that all of the cortical layers are in view of both **(A)** control (IFT88 f/f) and **(B)** mutants (IFTΔ/Δ). Cortical and CA1 layers are labeled (I, II/III, IV, V, VI, CA1). Hoechst nuclear stain is in blue. Scale bar is 100 µm.
(TIF)

File S3 **Cilia in the control IFT88^flox/flox^ adult hippocampus.** Immunofluorescence for the neuronal cilia marker adenylate cyclase III (red) in the wildtype hippocampus. Hoechst nuclear stain is in blue. Scale bar is 100 µm.
(TIF)

File S4 **Cilia in the IFT88^Δ/Δ^; Emx1-Cre mutant adult hippocampus.** Immunofluorescence for the neuronal cilia marker adenylate cyclase III (red) in the wildtype hippocampus. Hoechst nuclear stain is in blue. Scale bar is 100 µm.
(TIF)

File S5 **Loss of hippocampal and cortical primary cilia does not affect vision.** (**A**) Electroretinographs from control (Ift88^flox/flox^) and mutant (Ift88^Δ/Δ^) under dim, dark and light adapted conditions. (**B**) PCR genotyping from whole eye genomic DNA from Emx1 Cre positive IFT88^flox/flox^ (F/F) and IFT88^flox/wildtype^ (F/W) samples next to an Emx1 Cre cortex control Ift88^Δ/Δ^ (Δ/Δ).
(TIF)
