# Supplementary figures and images for: Hippocampal and Cortical Primary Cilia Are Required for Aversive Memory in Mice
Source: PLoS One. 2014 Sep 3;9(9):e106576. doi: 10.1371/journal.pone.0106576 (PMC4153651; doi:10.1371/journal.pone.0106576)

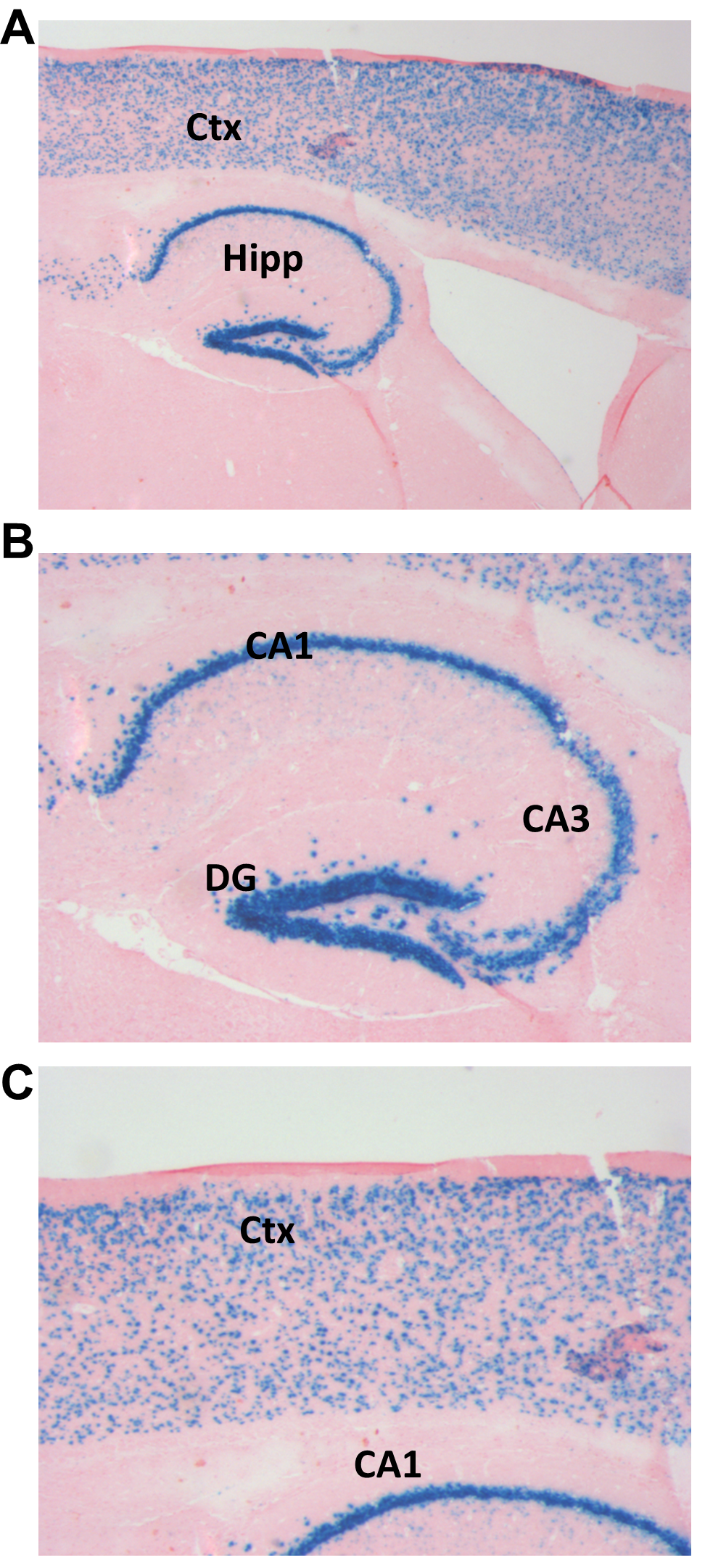

Supplement: File S1 — Cre reporter lacZ staining of brains sections shows the regions of cre activity. (A) Sagittal 20 µm thick brain section of the cortex (Ctx) and hippocampus (Hipp) stained for cre reporter LacZ activity in blue. (B) Image of the hippocampus showing the CA1, CA3 and dentate gyrus (DG). (C) Image of the cortex (Ctx) and CA1 of the hippocampus. (TIF) [file pone.0106576.s001.tif]

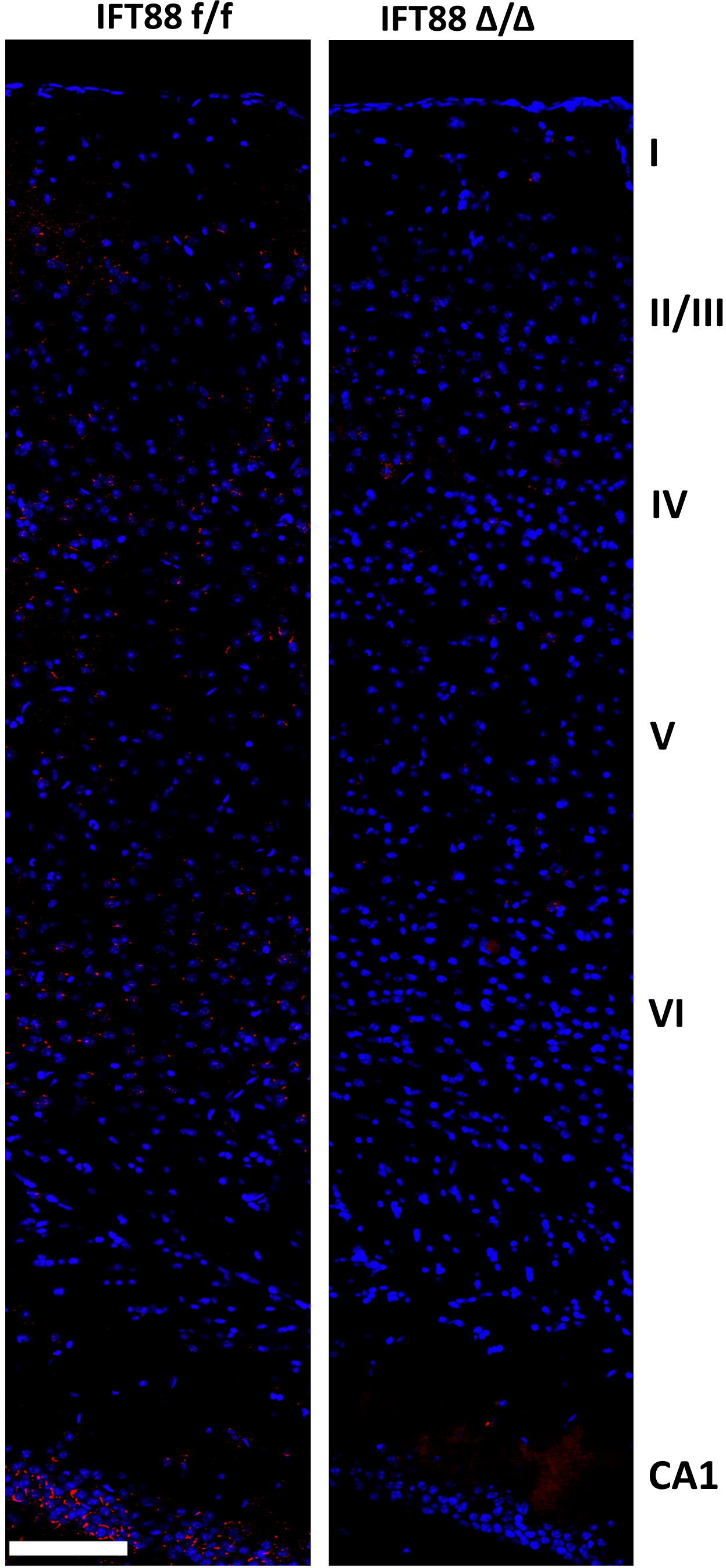

Supplement: File S2 — Conditional loss of Cilia in the adult cortex. Immunofluorescence for the neuronal cilia marker adenylate cyclase III (red) in the cortex (Ctx). (A and B) Stitching of several 20X images such that all of the cortical layers are in view of both (A) control (IFT88 f/f) and (B) mutants (IFTΔ/Δ). Cortical and CA1 layers are labeled (I, II/III, IV, V, VI, CA1). Hoechst nuclear stain is in blue. Scale bar is 100 µm. (TIF) [file pone.0106576.s002.tif]

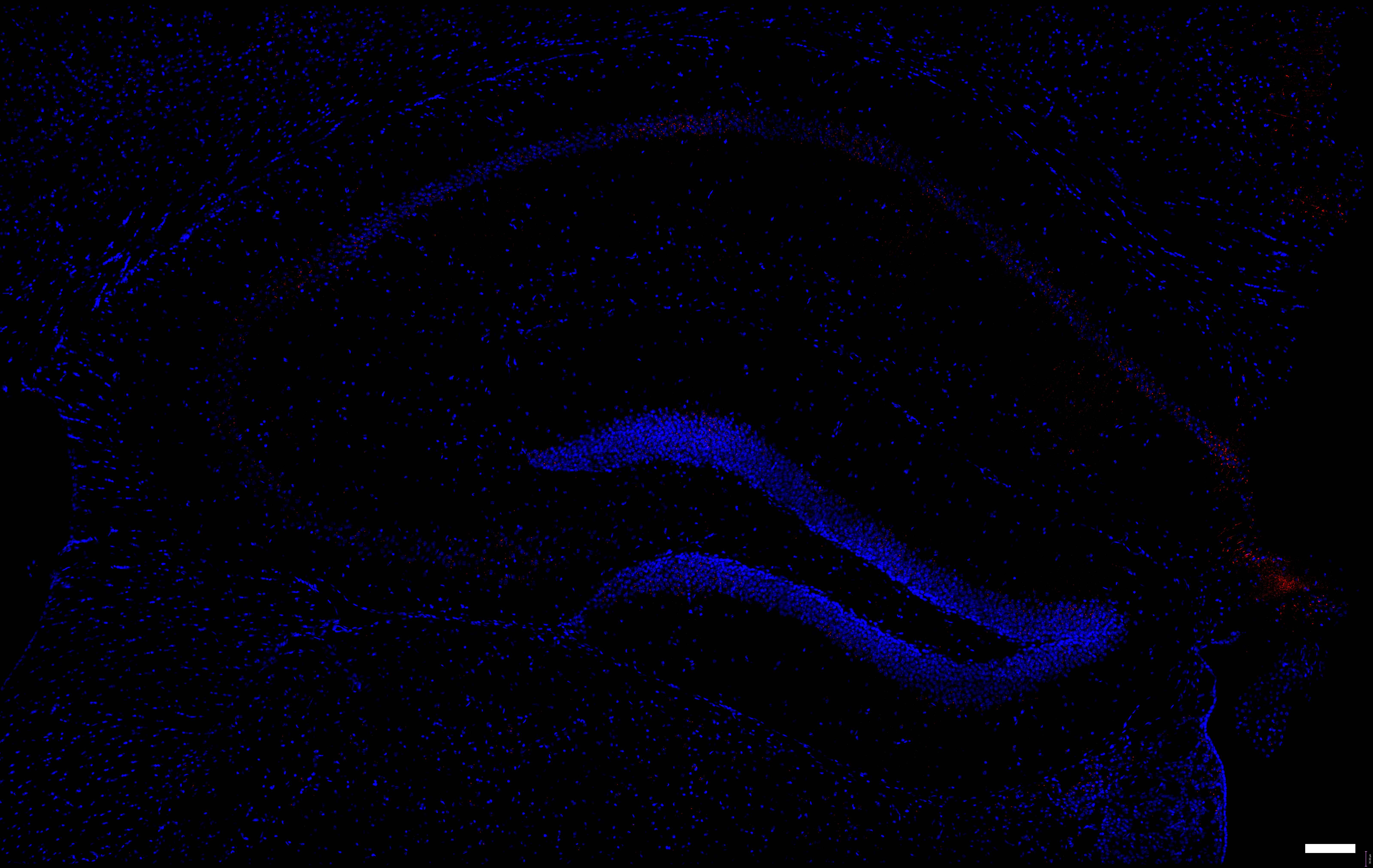

Supplement: File S3 — Cilia in the control IFT88flox/flox adult hippocampus. Immunofluorescence for the neuronal cilia marker adenylate cyclase III (red) in the wildtype hippocampus. Hoechst nuclear stain is in blue. Scale bar is 100 µm. (TIF) [file pone.0106576.s003.tif]

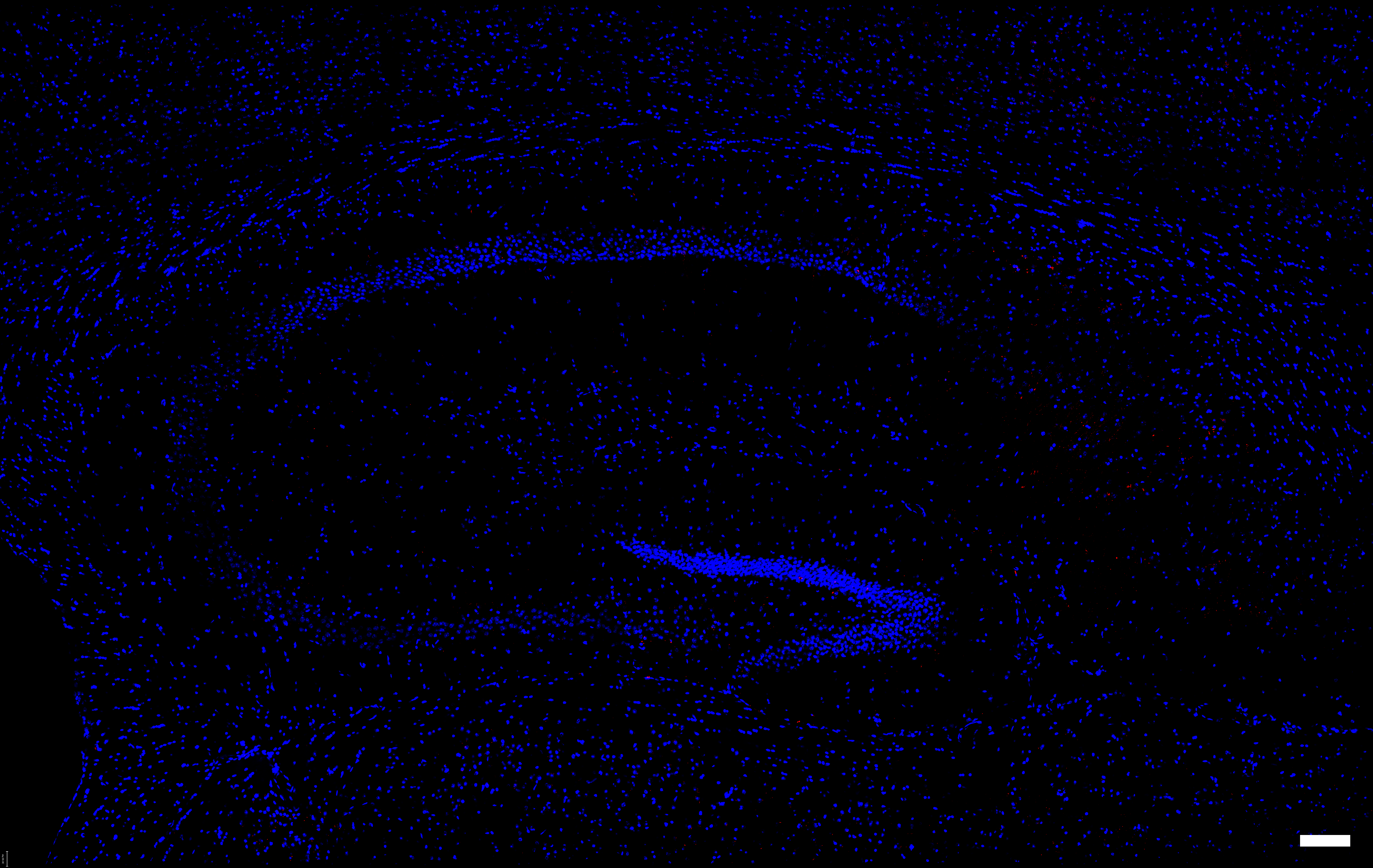

Supplement: File S4 — Cilia in the IFT88Δ/Δ; Emx1-Cre mutant adult hippocampus. Immunofluorescence for the neuronal cilia marker adenylate cyclase III (red) in the wildtype hippocampus. Hoechst nuclear stain is in blue. Scale bar is 100 µm. (TIF) [file pone.0106576.s004.tif]

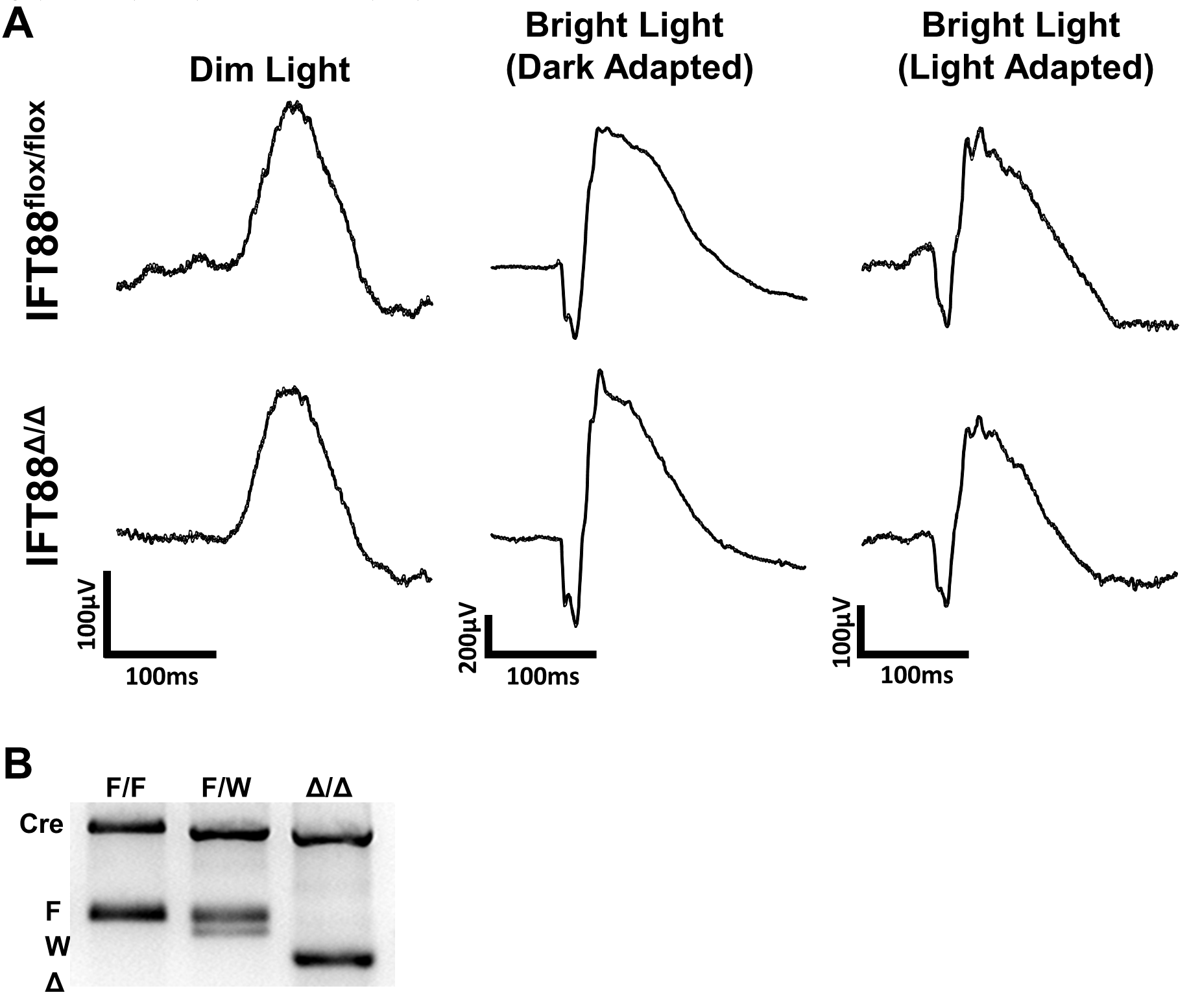

Supplement: File S5 — Loss of hippocampal and cortical primary cilia does not affect vision. (A) Electroretinographs from control (Ift88flox/flox) and mutant (Ift88Δ/Δ) under dim, dark and light adapted conditions. (B) PCR genotyping from whole eye genomic DNA from Emx1 Cre positive IFT88flox/flox (F/F) and IFT88flox/wildtype (F/W) samples next to an Emx1 Cre cortex control Ift88Δ/Δ (Δ/Δ). (TIF) [file pone.0106576.s005.tif]
